# LT1-3, a Slit2-Derived Peptide, Exhibits Anti-Tumor Activity and Improves Cisplatin Therapy

**DOI:** 10.3390/cells14211654

**Published:** 2025-10-22

**Authors:** Ting-Chien Wu, Chen-Yi Liao, Yu-Ying Lin, Shu-Ming Chuang, Szu-Yu Liu, Chi-Hsiang Wang, Shang-Er Su, Siang-Wei Wu, Ling-I Wang, Wei-Ting Chen, Sheng-Wen Cheng, Yu-Tang Huang, Yao-Bin Zheng, Cheng-Yen Chuang, Feng-Di Lung, Jinghua Tsai Chang

**Affiliations:** 1 Institute of Medicine, Chung Shan Medical University, 110, Sec. 1, Chien-Kuo N. Road, Taichung City 402306, Taiwan; alien11241@gmail.com (T.-C.W.); firmsky02@yahoo.com.tw (C.-Y.L.); glucosewater@yahoo.com.tw (S.-E.S.); eric10743@yahoo.com.tw (S.-W.W.); mastyehwang@gmail.com (L.-I.W.); jc610067@gmail.com (W.-T.C.); van811217@gmail.com (S.-W.C.); 2 Institute of Medical and Molecular Toxicology, Chung Shan Medical University, 110, Sec. 1, Chien-Kuo N. Road, Taichung City 402306, Taiwan; iamyuying@gmail.com (Y.-Y.L.); yavo7437@hotmail.com (S.-M.C.); jean750509@yahoo.com.tw (S.-Y.L.); chi.hsiang0523@gmail.com (C.-H.W.); 3 Department of Medical Laboratory and Biotechnology, Chung Shan Medical University, 110 Sec. 1, Chien-Kuo N. Road, Taichung City 402306, Taiwan; and6969dna@gmail.com (Y.-T.H.); ben24969309@gmail.com (Y.-B.Z.); 4 Division of Thoracic Surgery, Taichung Veterans General Hospital, No. 1650, Sec. 4, Taiwan Boulevard, Taichung City 407219, Taiwan; 5 Department of Chemistry, Tunghai University, Taichung, No. 1727, Sec. 4, Taiwan Boulevard, Taichung City 407224, Taiwan; 6 CSMU Lung Cancer Research Center, Chung Shan Medical University, 110, Sec. 1, Chien-Kuo N. Road, Taichung City 402306, Taiwan; 7 Divisions of Medical Oncology and Pulmonary Medicine, Chung Shan Medical University Hospital, 110, Sec. 1, Chien-Kuo N. Road, Taichung city 402306, Taiwan

**Keywords:** LT1-3, Slit2, p53, *JNK1*, Cisplatin, APR246

## Abstract

**Highlights:**

- The LT1-3 peptide inhibits proliferation and invasion of lung cancer cells while being non-toxic to normal cells.
- LT1-3 enhances the efficacy of cisplatin and appears to reduce its side effects.
- LT1-3 is suitable for combination with cisplatin as a first-line treatment for lung cancer and can be added to immunotherapy and platinum doublet therapy.
- LT1-3 can be continued as maintenance therapy, offering a promising new approach to improve treatment outcomes and patient quality of life.

**Abstract:**

The Slit2/Robo signaling pathway acts as a tumor suppressor in various cancers. This study identified an 8-amino acid peptide, LT1-3, derived from the Slit2 LamG domain, and demonstrated its ability to inhibit lung cancer cell proliferation and invasion independently of Robo receptors. Notably, LT1-3 was non-toxic to normal cells (Beas-2B, MRC5, and HUVECs). Combination treatment of LT1-3 and cisplatin synergistically inhibited the proliferation of lung cancer cells (CL1-5, A549, H1355, H460, H23, H661), but had no inhibitory effect on H1299 and H1975. Furthermore, combination therapy prolonged the median survival of tumor-bearing immunodeficient nude mice from 27.5 days (control) to 37.5 days (LT1-3 or cisplatin) and further to 47.5 days (LT1-3/cisplatin combination). The tumor suppressor *TP53* positively influences LT1-3-mediated proliferation inhibition, while *MAPK8 (JNK1)* and *PRKACA* (*PKA*) have been identified as negative regulators. With the exception of the p53R273 variants, most *TP53* mutants retained their function in this context. The p53 reactivator APR-246 restores sensitivity of p53R273H-expressing cells to LT1-3. JNK inhibition sensitizes p53-deficient or p53R273H-expressing cells to LT1-3-mediated proliferation inhibition. LT1-3, alone or in combination with a JNK inhibitor, enhances cisplatin efficacy, even in the presence of p53 mutations. Therefore, LT1-3 possesses multifunctional antitumor properties, directly inhibiting tumor cells and enhancing the efficacy of cisplatin, without causing toxicity to normal cells. Combining LT1-3 with cisplatin holds promise as a first-line therapy for lung cancer, while LT1-3 alone may be suitable for maintenance therapy.

## 1. Introduction

Slits are secreted glycoproteins that interact with Robo receptors to regulate axon pathfinding in the central [1,2] and sensory nervous systems [3,4]. Beyond neural development, Slit2/Robo1 signaling suppresses mammary branching [5] and plays crucial roles in the development of kidneys [6], heart [7], and lungs [8]. Mammals produce three Slits (Slit1-Slit3) and four Robo receptors (Robo1-Robo4) [9]. Human Slit2 comprises four leucine-rich (LRR, D1-D4) domains, nine epidermal growth factor-like (EGF 1-9) domains, a laminin-G (LamG)-like domain, and a C-terminal cysteine-rich knot-like domain. Slit2 can undergo cleavage between the EGF5 and EGF6 domains, generating N-terminal (Slit2-N, 140 kDa) and C-terminal fragments (Slit2-C, 55-60 kDa) [10]. The D2 region of full-length Slit2 and Slit2-N directly interacts with the IgG1 and IgG2 domains of Robo1 [11].

Recently, Slit2/Robo signaling has garnered attention in cancer research, particularly regarding cell proliferation, migration, and angiogenesis. However, conflicting results exist across various tumor types. While Slit2/Robo plays a tumor-suppressing role in breast cancer [12], lung cancer [13], glioma [14], esophageal carcinoma [15], and colorectal cancer [16] cells, it exhibits cancer-promoting activity in specific contexts [17,18,19]. For instance, the Slit2/Robo1 axis has been linked to cancer proliferation via the Warburg effect in osteosarcoma [20], and it induces epithelial–mesenchymal transition (EMT) in colorectal epithelial cells [21] by associating Src and E-cadherin with the Robo1 cytoplasmic domain.

We have identified two *Slit2* exon 15 splicing variants: wild-type *Slit2* (*Slit2*-WT) with intact exon 15, and a splicing variant, *Slit2*-ΔE15, lacking exon 15. Exon 15 of *Slit2* encodes 8 amino acids (AKEQYFIP) situated at the end of the D2 domain. Our previous work demonstrated that *Slit2*-WT inhibits lung cancer cell proliferation, while *Slit2*-ΔE15 suppresses both proliferation and invasion [22]. Here, we aim to identify the domains responsible for these functions. We identified an eight-amino acid peptide (LT1-3) within the C-terminal region of both Slit2-WT and Slit2-ΔE15, exhibiting dual functionality in inhibiting lung cancer cell proliferation and invasion. Notably, LT1-3’s action is Robo-independent, potentially circumventing the conflicting roles of full-length Slit2 in cancer cells’ Slit/Robo signaling. Our study demonstrates LT1-3’s multifunctionality, its intrinsic antitumor activity, its enhancement of cisplatin efficacy in many *TP53* mutant lung cancer cells, its reduction in cisplatin-induced side effects, and its non-toxicity to normal cells. Consequently, combining LT1-3 with cisplatin emerges as a promising first-line anti-lung cancer therapy and maintenance therapy.

## 2. Materials and Methods

### 2.1. Animal Studies

Female CAnN. Cg-*Foxn1^nu^*/CrlBltw mice were acquired from Biolasco Taiwan Co., Ltd. (Taipei, Taiwan). For the xenograft tumor model, 2 × 10^6^ CL1-5 cells with 20% Matrigel (Corning, New York, NY, USA) were subcutaneously injected into 7-week-old nude mice. Three days post-inoculation, mice were subcutaneously administered 10 nmol (0.58 mg/kg), 20 nmol (1.16 mg/kg), or 30 nmol (1.74 mg/kg) of F-LT1-3 peptides in 0.5% DMSO near the cancer cell injection site, five times per week for 18 days. Starting on day 19, F-LT1-3 was dissolved in 0.5% γ-cyclodextrin (γ-CD) and administered daily. Tumor volume was measured twice per week using a Vernier caliper and calculated as follows: tumor size (mm^3^) = [length × (width)^2^]/2. The mice were sacrificed after 40 days.

To assess the efficacy of F-LT1-3 in combination with cisplatin, we subcutaneously injected CL1-5 cancer cells into mice’s upper back and injected F-LT1-3 subcutaneously into their lower back. When tumors reached 50–100 mm^3 in 80% of mice, mice were randomly assigned to five groups before treatment initiation: (1) control (*n* = 6), (2) cisplatin alone (4 mg/kg (Fresenius Kabi Oncoloy Ltd., Solan, India) intraperitoneal injection weekly for 4 weeks) (*n* = 8), (3) F-LT1-3 alone (1.16 mg/kg subcutaneous injection daily) (*n* = 7), (4) maintenance treatment (2 cycles cisplatin followed by cisplatin and F-LT1-3 co-treatment for 2 weeks, then daily F-LT1-3) (*n* = 8), and (5) combination therapy (four cisplatin and F-LT1-3 co-treatments followed by F-LT1-3 treatment) (*n* = 8). Survival was analyzed using the Kaplan-Meier method. All animal study procedures were approved by the Institutional Animal Care and Use Committee (IACUC) of Chung Shan Medical University (CSMU-IACUC-1894).

### 2.2. Cell Lines and Cell Culture

A549 (RRID:CVCL_0023), H1299 (RRID:CVCL_0060), H1975 (RRID:CVCL_1511), H1355 (RRID:CVCL_1465), H460 (RRID:CVCL_0459), H23 (RRID:CVCL_1547) and H661 (RRID:CVCL_1577) human lung cancer cells, MRC5 (RRID:CVCL_0440) human fetal lung fibroblast cells and HUVECs (Human umbilical vein endothelial cells: RRID:CVCL_0060) were obtained from Bioresource Collection and Research Center (BCRC, Hsinchu, Taiwan). Beas2B (RRID:CVCL_0168) was acquired from ATCC (Manassas, VA, USA). The CL1-0 (RRID:CVCL_3871) and CL1-5 (RRID:CVCL_D521) lung adenocarcinoma cell lines were kindly provided by Dr. P-C Yang (Department of Internal Medicine, National Taiwan University, Taipei, Taiwan). CL1-5, H661, H1355, and H23 cells were cultured in RPMI-1640 (Gibco, Thermo Fisher Scientific, Waltham, MA, USA). H1975 and H460 cells were cultured in RPMI-1640 with 10 mM HEPES. A549 and H1299 cells were cultured in DMEM (Gibco). MRC5 cells were maintained in MEM (Gibco). HUVECs were cultured in 0.2% gelatin-coated dishes with Medium 199 (Gibco) supplemented with heparin and endothelial cell growth supplement. All growth media were supplemented with 10% fetal bovine serum (FBS) (Gibco), except for Beas-2B, which was cultured with serum-free LHC-9 medium (Gibco). Cells were cultured at 37 °C with 5% CO_2_.

### 2.3. Cell Transfection and Stable Clone Selection

We performed transient and stable transfections using Lipofectamine 2000 (Invitrogen, Thermo Fisher Scientific) following the manufacturer’s protocol. For RNA interference, we used 100 pmol of si-*Robo1*, si-*Robo4*, si-*TP53*, 5 pmol of si-*PKAca*, or 12.5 pmol of si-*JNK1* for cell transfection. (Sequences of the siRNAs are listed in Supplementary Table S1). Stable cell lines were generated by culturing cells in media containing 500 μg/mL G418 (Sigma-Aldrich, Merck, Burlington, MA, USA) for 3–4 weeks.

### 2.4. Cell Proliferation Assay

Cell proliferation rates were assessed using the cell counting method. Cells were seeded in 12-well plates with the appropriate culture medium. After 16 h of incubation, cells were treated with LT1-3 or LT1-3 combined with cisplatin for 24 h. Cells were then collected, stained with trypan blue (Gibco), and quantified using a hemocytometer. The number of cells measured 16 h after seeding was designated as N_0_, and the number measured 24 h after drug treatment was designated as N_24_. Fold changes were calculated using the formula: Fold change = (N_24_ − N_0_)/N_0_.

### 2.5. Cloning and PCR-Based Site-Directed Mutagenesis

*Slit2*-WT and *Slit2*-ΔE15 cDNA constructs were cloned from CL1-0 lung adenocarcinoma cells as described previously [22]. Wild-type *TP*53 and mutant *TP*53 cDNAs were cloned from various lung cancer cell lines. We generated EGF6-LamG- or EGF7-9 domain-deleted *Slit2*-ΔE15, and p53R273 mutations using two-stage PCR-based site-directed mutagenesis. Overlapping complementary primers (forward mutant primer (F’-mp) and reverse mutant primer (R’-mp)) containing specific target deletions or mutations were designed (primers used for mutagenesis are listed in Supplementary Table S2). The first-stage PCR amplified the 5′ sequence of the mutated fragment with a forward upstream primer (F’-up) and R’-mp, and the 3′ sequence of the mutated fragment with an F’-mp and a reverse downstream primer (R’-dp). The second-stage PCR amplified the full-length PCR fragment after gel purification and mixing the mutated 5′- and 3′-PCR fragments, using F’-up and R’-dp primers. The amplified full-length fragment was then cleaved, cloned into an expression vector, and sequenced to verify the success of mutagenesis. *Slit2*-N- and *Slit2*-C-terminal fragments were prepared from *Slit2*-WT and amplified using PCR for cloning.

### 2.6. Western Blot Analysis

Western blot analysis was conducted as described previously [23]. Deletion or truncation of *Slit*2-ΔE15 constructs was detected with an anti-myc antibody (#05-419, Sigma-Aldrich), and ectopic expression of p53 was detected with anti-p53 antibody (#2527, Cell Signaling Technology, Danvers, MA, USA).

### 2.7. Quantitative Reverse Transcription–PCR

Total RNA (2 μg) was extracted from cell lines using TRIzol (Invitrogen) and treated with RQ1 DNase (Promega, Madison, WI, USA). cDNA synthesis used M-MLV reverse transcriptase (Promega), and Real-time PCR employed FastStart Universal SYBR Green Master Mix (ROX) (Bio-Rad, Hercules, CA, USA). Primer sequences are listed in Supplementary Table S3.

### 2.8. Wound Healing and Invasion Assays

Wound healing assays and invasion assays were performed to determine cell motility as described [22]. For the invasion assay, a Boyden chamber with a polycarbonate membrane (8-μm pore size, GVS Filter Technology, Bologna, Italy) coated with 1 μg/mL Matrigel (Becton Dickinson, Franklin Lakes, NJ, USA) was used [22]. CL1-5 cells (3 × 10^4^ cells/well) were seeded into the upper chamber, and invading cells were counted after 20 h under a light microscope.

### 2.9. Statistical Analysis

Data were analyzed using PASW Statistics 18 software (IBM, Armonk, NY, USA) and are presented as means ± standard deviations. Proliferation and invasion assays were performed at least three times, with representative figures presented. Statistical significance between groups was determined using Student’s *t* test or one-way ANOVA. For significant ANOVA value, Scheffe’s post hoc test was used for comparisons. Mean values with the same letter are not significantly different (*p* > 0.05). Survival analysis for mice treated with LT1-3 and cisplatin was performed using the Kaplan–Meier method. *p* values of <0.05 (*); <0.01 (**); <0.001 (***) were considered statistically significant.

## 3. Results

### 3.1. Effects of Slit2-N- and Slit2-C-Terminal Fragments on Lung Cancer Cell Proliferation and Invasion

**Figure 1:**
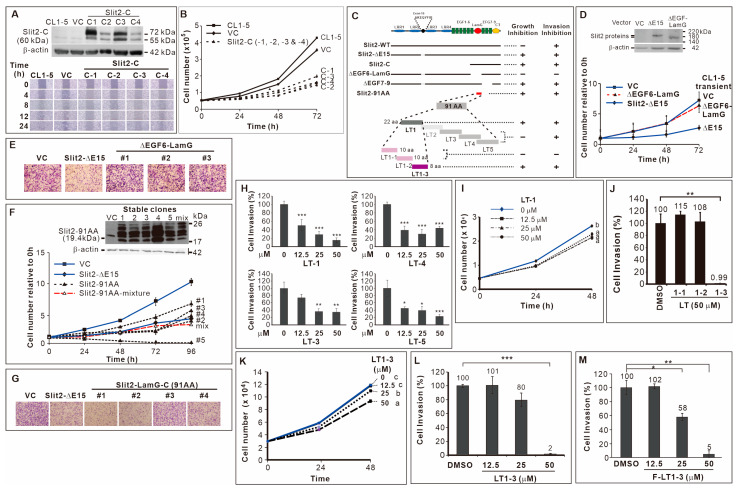
Identification of F-LT1-3 peptide. (**A**) CL1-5 clones stably expressing *Slit2*-C terminal domain showed reduced motility. (**B**) These *Slit2*-C stable clones also inhibited cell proliferation (*n* = 3). (**C**) Schematic diagram summarizing the deletion mutants, truncation mutants, and peptides used in this study. (**D**) Transient expression of *Slit2*-ΔE15/ΔEGF6-LamG failed to inhibit the proliferation of CL1-5 cells (*n* = 3). (**E**) When compared with the *Slit2*-ΔE15 stable clone, stable clones expressing *Slit2*-ΔE15/ΔEGF6-LamG lost the ability to inhibit cell invasion. CL1-5/*Slit2*-91AA stable clones inhibited cell proliferation (*n* = 3) (**F**) and invasion (**G**). (**H**) The LT-1, LT-3, LT-4 and LT-5 peptides inhibited invasion of CL1-5 cells (*n* = 3). (**I**) The LT-1 peptide inhibited the proliferation of CL1-5 cells. (**J**) The LT1-1, LT1-2 and LT1-3 peptides were derived from the LT-1 peptide. LT1-3, but not LT1-1 or LT1-2, inhibited CL1-5 cell invasion (*n* = 3). (**K**) LT1-3 also showed inhibitory effects on proliferation. (**L**) Dose effect of LT1-3 on the inhibition of cell invasion. (**M**) Fmoc-modified LT1-3 (Fmoc-LT1-3) showed higher inhibitory activity than did LT1-3 on cell invasion at a concentration of 25 mM (compare L & M, *n* = 3). All of the data are shown as the mean ± SD. Statistical significance was calculated using a two-tailed unpaired Student’s *t* test; or calculated using one-way ANOVA with Scheffe’s multiple comparison test (**I**,**K**). Data annotated with the same letter were not significantly different. * *p* < 0.05, ** *p* < 0.01, *** *p* < 0.001.

We identified two splicing variants of *Slit2* at exon 15; *Slit2*-ΔE15 inhibited the proliferation and invasion/motility of CL1-5 lung cancer cells, while *Slit2*-WT solely inhibited invasion/motility activity [22]. To pinpoint the domain of Slit2 responsible for invasion inhibition, we used *Slit2-*WT as a template and performed PCR to amplify *Slit2-*N and *Slit2-*C fragments (fused to preprotrypsin for secretion), subcloning them into a pcDNA vector to create stable clones in CL1-5 cells. Since *Slit2*-WT only inhibited invasion and the *Slit2*-N-terminal fragment was known to inhibit axon guidance [10], we hypothesized that *Slit2*-N, but not *Slit2*-C, inhibits cell migration. However, results from a wound healing assay showed inconsistent effects of *Slit2*-N stable clones on cell migration (Supplementary Figure S1A). Notably, all CL1-5 clones stably expressing *Slit2*-C displayed reduced motility (Figure 1A). Moreover, *Slit2*-C inhibited cell proliferation (Figure 1B), while *Slit2*-N had no effect on cell proliferation (Supplementary Figure S1B).

### 3.2. 91 Amino Acid-Long LamG Domain (91 AA) Inhibits Lung Cancer Cell Proliferation and Invasion

Since *Slit2*-ΔE15 inhibited cell proliferation and invasion and because it includes the *Slit2*-C-terminal fragment, we speculated that the Slit*2*-C-terminal domain is involved in these effects. Consequently, we deleted the EGF6-LamG or EGF7-9 domain in the C-terminal (Figure 1C), naming the constructs ΔEGF6-LamG and ΔEGF7-9, respectively. We found that two stable clones of ΔEGF6-LamG (Supplementary Figure S1C) and transient transfection of CL1-5 with ΔEGF6-LamG abolished the inhibitory effect of *Slit2*-ΔE15 on cell proliferation (Figure 1D). However, all the ΔEGF7-9 stable clones retained a slow proliferation rate (Supplementary Figure S1D). Next, we examined these deletion constructs’ role in cell invasion. The results showed that CL1-5/ΔEGF6-LamG stable clones exhibited high invasive activity (Figure 1E). However, the invasive activity of all CL1-5/ΔEGF7-9 stable clones was as low as that of CL1-5/*Slit2*-ΔE15 (Supplementary Figure S1E). Finally, we subcloned a 91 amino acid (91-AA) region of the LamG domain (Slit2-91AA, Figure 1C) and fused it with the signal peptide in preprotrypsin, selecting CL1-5 clones stably expressing the Slit2-91AA protein fragment encoding sequence. The results showed that 91-AA segment of the Slit2 protein sufficiently inhibited the proliferation (Figure 1F) and invasion (Figure 1G) of CL1-5 cells.

### 3.3. Inhibitory Effect of the 8-Amino Acid Peptide LT1-3 in the LamG-C Domain on Cancer Cell Proliferation and Invasion

Subsequently, we generated five partially overlapping peptides LT-1, LT-2, LT-3, LT-4, and LT-5 (Figure 1C, sequences listed in Supplementary Table S4) to elucidate the inhibitory mechanisms of the 91 AA-long fragment in the Slit2-LamG domain on cell proliferation and invasion. Due to cyclization, LT-2 peptide synthesis failed. The LT-1, LT-3, LT-4, and LT-5 peptides all exhibited inhibitory effects on CL1-5 cell invasion (Figure 1H). Among them, only LT-1 peptide also inhibited cell proliferation (Figure 1I, Supplementary Figure S2A). We further synthesized three partially overlapping peptides based on the LT-1 sequence, resulting in LT1-1, LT1-2, and LT1-3 (Figure 1C, Supplementary Table S4). Notably, the LT1-3 peptide inhibited the invasion (Figure 1J) and proliferation (Figure 1K) of CL1-5 lung cancer cells, whereas LT1-1 and LT1-2 did not have these effects (Supplementary Figure S2B). To enhance LT1-3 stability, we modified it using an N-terminal fluorenylmethyloxycarbonyl (Fmoc) group to prevent degradation by potential exopeptidases. This modification enhanced the inhibitory activity of LT1-3 (F-LT1-3) on cell invasion at a concentration of 25 μM (compare Figure 1L and Figure 1M). Thus, we identified an 8-amino acid peptide, F-LT1-3, from the LamG domain of Slit2 that exhibited an inhibitory effect on the proliferation and invasion of CL1-5 cells.

### 3.4. F-LT1-3 Peptide Inhibits Tumor Progression, Reduces Cisplatin-Induced Side Effects, and Prolongs Animal Survival

**Figure 2:**
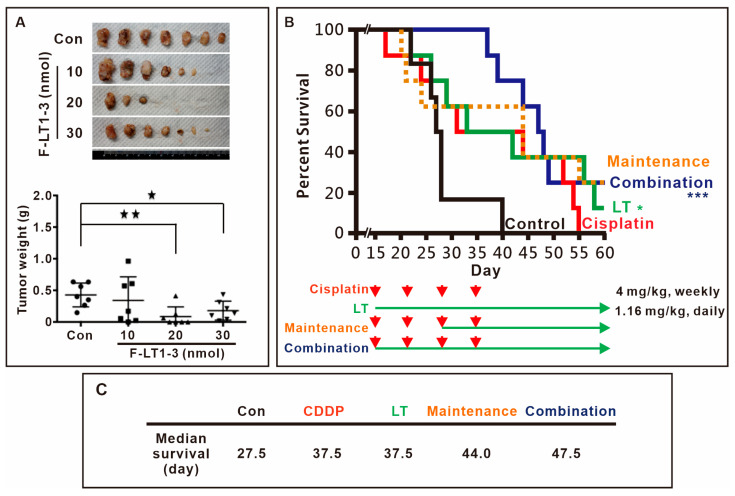
Fmoc-LT1-3 and cisplatin cotreatment prolongs animal survival. (**A**) Nude mice were subcutaneously injected with CL1-5 lung cancer cells. Three days after injection, the mice were subcutaneously injected with 10 nmol (0.58 mg/kg), 20 nmol (1.16 mg/kg), or 30 nmol (1.74 mg/kg) of F-LT1-3 peptide near the site of the cancer cell injection. The mice were sacrificed on day 40, and tumors were removed and weighed. In the F-LT1-3 treated group, tumors were not found in 4 out of 7 mice. (**B**) The survival rate of mice was determined on the basis of the following groups: (i) control (empty vehicle injection), (ii) cisplatin treatment (4 mg/kg, intraperitoneal injection, weekly for 4 weeks, red arrows.), (iii) F-LT1-3 treatment (1.16 mg/kg subcutaneous injection daily), (iv) maintenance treatment (cisplatin for 2 weeks followed by cisplatin/F-LT1-3 cotreatment for 2 weeks and then F-LT1-3 alone until the end) (v) cisplatin/F-LT1-3 cotreatment (cotreatment for 4 weeks followed by F-LT1-3 to the end). (**C**) The median survival of the control group was 27.5 days that of the cisplatin-only and F-LT1-3-only groups were 37.5 and 37.5 days, respectively, that of the maintenance group was higher at 44 days, and that of the combination group reached 47.5 days. All of the data are shown as the mean ± SD. Statistical significance was calculated using a two-tailed unpaired Student’s *t* test. * *p* < 0.05, ** *p* < 0.01, *** *p* < 0.001.

We then assessed the in vivo efficacy of F-LT1-3 peptide inhibition using xenograft mouse models. The results demonstrated that 1.16 mg/kg (20 nmol) F-LT1-3 peptide significantly reduced tumor formation (Figure 2A). Furthermore, we investigated the effects of F-LT1-3 in combination with cisplatin in an animal model. Mice treated with 4 mg/kg of cisplatin exhibited weakness and thin skin and died spontaneously after cisplatin injection (the cisplatin-only and maintenance groups) (Figure 2B). However, mice treated with a combination of cisplatin and F-LT1-3 displayed activity levels similar to those in the control and F-LT1-3-only groups and exhibited normal skin appearance. Ultimately, the median survival time of the control group was 27.5 days, while both the cisplatin-only and F-LT1-3-only groups had a median survival time of 37.5 days. In comparison, the maintenance group survived for 44 days, and the combination group showed the highest median survival time at 47.5 days (Figure 2C). Despite similar median survival times in the cisplatin-only and F-LT1-3-only groups, the cisplatin-treated mice exhibited severe illness, inactivity, and thin skin, whereas those treated with F-LT1-3 alone or in combination with cisplatin remained active with normal skin. Therefore, we concluded that F-LT1-3 not only inhibited tumor growth but also appeared to reduce the severity of cisplatin-induced side effects and prolonged animal survival.

### 3.5. PEGylation Enhances the Solubility of F-LT1-3 While Retaining Its Proliferation and Invasion Inhibition Activity

As F-LT1-3 is a hydrophobic peptide, we enhanced its solubility by adding polyethylene glycol (PEG) to either its N- or C-terminus. When three PEG molecules (PEG3) were added to the N-terminus of LT1-3 (PEG-LT1-3), the peptide’s inhibitory activity on cell invasion was eliminated (Supplementary Figure S3A), and its inhibitory activity on cell proliferation was reduced (Supplementary Figure S3B). Interestingly, when F-LT1-3 was modified with PEG3 at the C-terminus (F-LT1-3-PEG), it maintained its inhibitory effect on both cell proliferation and invasion (Supplementary Figure S3C–E).

### 3.6. F-LT1-3-PEG Inhibits Lung Cancer Cell Proliferation, but Not Normal Cell Proliferation

**Figure 3:**
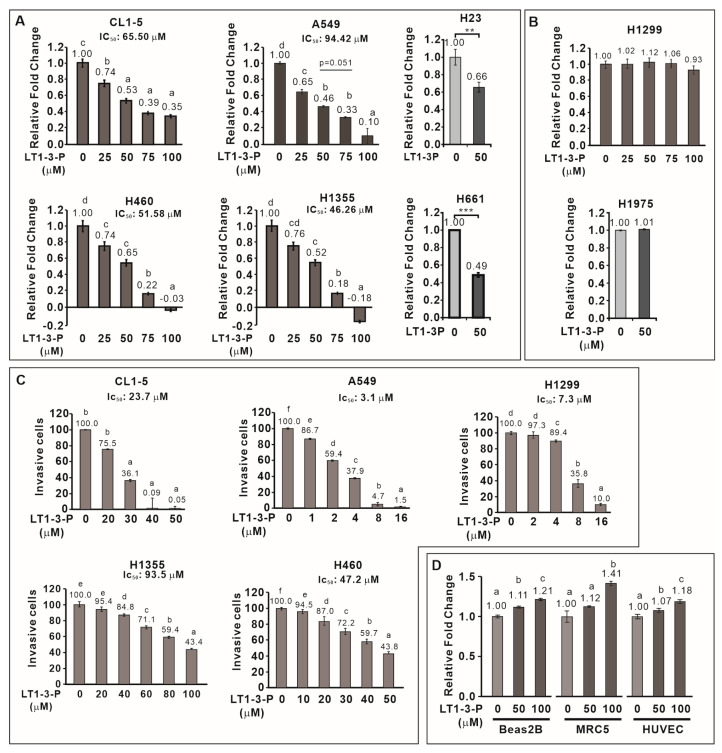
Effect of F-LT1-3-PEG on the proliferation and invasion inhibition of various lung cancer cell lines and normal cell lines. (**A**,**B**) Dose effect of F-LT1-3-PEG on the inhibition of lung cancer cell proliferation. (**C**) Dose effect of F-LT1-3-PEG on the inhibition of lung cancer cell invasion. (**D**) F-LT1-3-PEG did not inhibit the proliferation of Beas2B, MRC5, or HUVEC normal cell types. All of the data are shown as the mean ± SD. Statistical significance was calculated using one-way ANOVA with Scheffe’s multiple comparison test. Data annotated with the same letter were not significantly different. ** *p* < 0.01, *** *p* < 0.001.

We then examined the inhibitory effect of F-LT1-3-PEG on the proliferation of various human lung cancer cell lines and normal cells. F-LT1-3-PEG inhibited the proliferation of A549, CL1-5, H1355, H460, H23, and H661 lung cancer cell lines (Figure 3A) but not H1299 or H1975 cells (Figure 3B). Despite its inability against H1299 cells in proliferation inhibition, F-LT1-3-PEG effectively inhibited invasion across all tested cell lines, including H1299 cells (Figure 3C). Importantly, F-LT1-3-PEG did not inhibit the proliferation of two normal cells lines, MRC5 and Beas2B, or human umbilical vein endothelial cells (HUVECs) (Figure 3D).

### 3.7. TP53 Positively Regulates F-LT1-3-PEG-Mediated Inhibition of Lung Cancer Cell Proliferation

**Figure 4:**
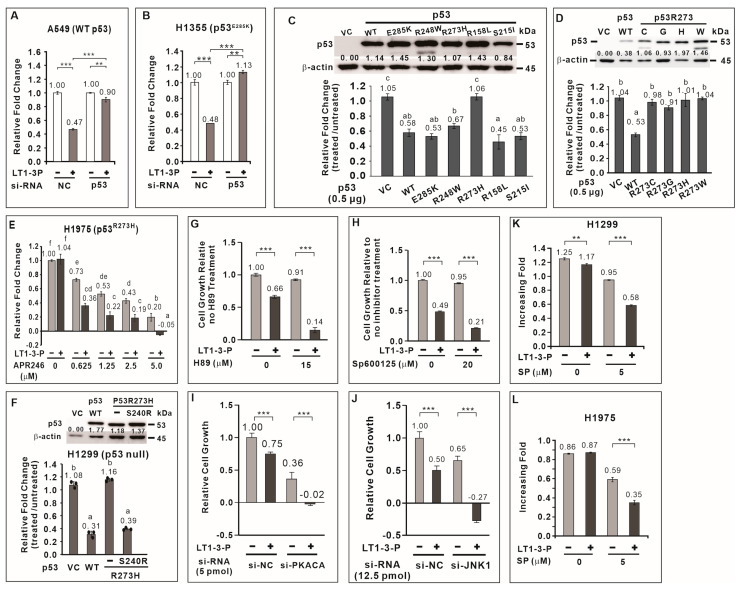
Role played by p53, PKA, and JNK1 in F-LT1-3-PEG-mediated inhibition of cell proliferation. Knocking down the expression of wild-type *TP53* (**A**) and p53E285K mutant p53 (**B**) using si-*TP*53 abolished F-LT1-3-PEG-mediated inhibition of A549 and H1355 cell proliferation, respectively, suggesting that p53 is important for F-LT1-3-PEG-mediated proliferation inhibition. (**C**) Effect of wild-type or mutant *TP*53 cDNA on restoring the sensitivity of H1299 cells to F-LT1-3-PEG-mediated growth inhibition. The relative change in the number of cells is reported as the fold change and was determined using the fold change of the number of treated cells divided by the fold change of the number of untreated cells. (**D**) All p53 mutants in which R273 was replaced with a C, G, H, or W residue did not mediate the inhibitory effect of F-LT1-3-PEG on cell proliferation. (**E**) The p53 reactivator APR-246 drove the switching of H1975 cell phenotype from resistant to sensitive, which responded to F-LT1-3-PEG inhibition of proliferation. (**F**) The p53R273H suppressor S240R restored the sensitivity of H1299 cells to growth inhibition mediated by F-LT1-3-PEG. (**G**,**H**) H89, a PKA inhibitor, and SP600125, a JNK inhibitor enhanced the proliferation inhibiting activity of F-LT1-3-PEG in CL1-5 cells. (**I**,**J**) Knocking down the expression of *PKACA* or *JNK1* using siRNAs also enhanced the inhibitory effect of F-LT1-3-PEG on CL1-5 cell proliferation. (**K**,**L**) The JNK inhibitor drove the F-LT1-3-PEG-resistant cell lines H1299 and H1975 to acquire a sensitive phenotype. All of the data are shown as the mean ± SD. Statistical significance was calculated using a two-tailed unpaired Student’s *t* test; or calculated using one-way ANOVA with Scheffe’s multiple comparison test (**C**–**F**). Data annotated with the same letter were not significantly different. ** *p* < 0.01, *** *p* < 0.001.

To elucidate the mechanism underlying F-LT1-3-PEG-mediated inhibition of lung cancer cell proliferation, we focused on *TP*53, a crucial tumor suppressor frequently mutated in lung cancer. Among lung cancer cells tested, H1299 cells lack functional p53, while A549 and H460 cells have wild-type p53. CL1-5, H1355, H23, H661, and H1975 cells carry the R248W, E285K, M246I, R158L, and R273H p53 mutants, respectively (Table 1). We hypothesized that p53 plays a critical role in the growth inhibitory effect of the peptide, with its effectiveness depending on the specific mutation. We found that knockdown of wild-type *TP*53 in A549 or p53^E285K^ in H1355 cells significantly attenuated the inhibitory effect of F-LT1-3-PEG on cell proliferation (Figure 4A,B). This suggests that *TP*53 positively regulates F-LT1-3-PEG-mediated inhibition of lung cancer cell proliferation.

### 3.8. Most p53 Mutants Retain the Ability to Mediate F-LT1-3-PEG-Induced Inhibition of Lung Cancer Cell Proliferation

We then investigated whether *TP*53 mutation affect F-LT1-3-PEG’s inhibitory effect on lung cancer cell proliferation. We cloned *TP*53 cDNA from wild-type *TP*53 and several *TP*53 mutant cell lines. Ectopic expression of wild-type p53 in H1299 cells restored F-LT1-3-PEG-mediated inhibition of cell proliferation (Figure 4C). Interestingly, ectopic expression of p53^E285K^, p53^R248W^, p53^R158L^, and p53^S215I^ mutants also restored F-LT1-3-PEG-mediated inhibition of cell proliferation to a similar extent as wild-type p53 (Figure 4C). However, the p53 R273 mutants (R273H, R273C, R273G, and R273W) failed to restore F-LT1-3-PEG-mediated inhibition of cell proliferation in H1299 cells (Figure 4D). These results indicate that most p53 mutants retain their ability to mediate F-LT1-3-PEG-mediated inhibition of cell proliferation, except for the p53 R273 mutant.

### 3.9. APR-246 Restores Sensitivity to F-LT1-3-Mediated Inhibition of Cell Proliferation in p53^R273H^-Expressing Cells

APR-246 (Eprenetapopt) is a small molecule that can restore wild-type conformation and function to some mutant p53 proteins, particularly the R273H mutant [24]. We found that APR246 treatment restored the sensitivity of H1975 cells expressing p53^R273H^ to F-LT1-3-PEG-meidated inhibition of cell proliferation (Figure 4E). Furthermore, studies have shown that S240R is a suppressor of p53^R273H^ [25,26]. Therefore, we introduce S240R into p53^R273H^ and found that p53^R273H/S240R^ restored the proliferation inhibitory activity of F-LT1-3-PEG in H1299 cells (Figure 4F). The above results indicate that restoring the structure or function of p53^R273H^ can restore the ability of p53^R273H^ to mediate F-LT1-3-PEG’s inhibitory effect on cell proliferation.

### 3.10. JNK1 and PKA Negatively Regulate F-LT1-3-PEG-Mediated Inhibition of Cell Proliferation

To further explore the signaling pathways involved in F-LT1-3-PEG-mediated inhibition of cell proliferation, we examined the roles of JNK1 and PKA, two kinases known to be involved in cell proliferation and apoptosis. We found that inhibition PKA activity by H89 or JNK activity by SP600125 enhanced F-LT1-3-PEG’s inhibitory activity in cell proliferation (Figure 4G,H). Knockdown of *PKA* or *JNK1* expression also enhanced F-LT1-3-PEG-mediated inhibition of CL1-5 cell proliferation (Figure 4I,J). Surprisingly, inhibition of JNK1 activity in H1299 (Figure 4K) and H1975 (Figure 4L) cells sensitized both cell lines to F-LT1-3-PEG-mediated inhibition of cell proliferation. Conversely, treatment with an NFκB inhibitor (Bay11-7082), MEK inhibitor (U0126), PI3K inhibitor (LY294002), or p38 inhibitor (SB203580) reduced F-LT1-3-PEG’s proliferation-inhibiting activity (Supplementary Figure S4). These results indicate that JNK1 and PKA negatively regulate F-LT1-3-PEG-mediated inhibition of lung cancer cell proliferation.

### 3.11. F-LT1-3-PEG Enhances Cisplatin Efficacy in Lung Cancer Cells

**Figure 5:**
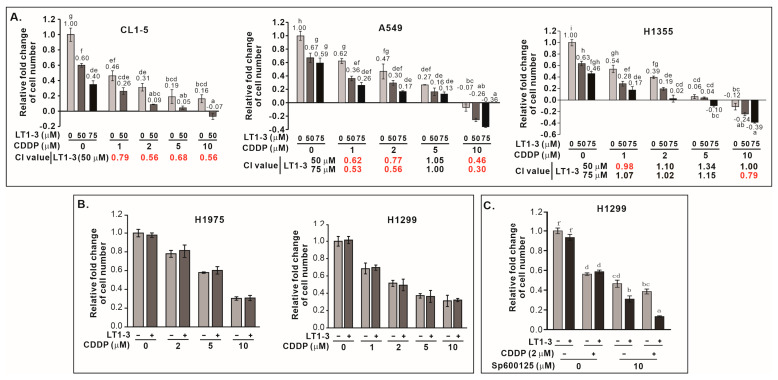
F-LT1-3-PEG enhances the cytotoxicity of cisplatin in lung cancer cell lines. (**A**) The inhibition of cell proliferation was enhanced when cells were treated with different concentrations of cisplatin and F-LT1-3-PEG. Combination index (CI) values were calculated to characterize synergism (CI < 1, marked in red), additive effect (CI = 1) and antagonism (CI > 1) at different dose combinations. (**B**) Cell proliferation inhibition induced by cotreatment with cisplatin and F-LT1-3-PEG was not additive or synergistic in H1299 and H1975 cells. (**C**) The JNK inhibitor SP600125 successfully restored the enhanced inhibitory effect of cotreatment with cisplatin and F-LT1-3-PEG on H1299 cell proliferation. All of the data are shown as the mean ± SD. Statistical significance using a two-tailed unpaired Student’s *t* test; or calculated using one-way ANOVA with Scheffe’s multiple comparison test (**A**,**C**). Data annotated with the same letter were not significantly different.

Given that p53 mutations often confer resistance to chemotherapy, we investigated whether F-LT1-3-PEG could enhance cisplatin efficacy in lung cancer cells with p53 mutation. Combining F-LT1-3-PEG and cisplatin significantly boosted proliferation inhibition in CL1-5, A549, and H1355 (Figure 5A) and other cell lines (Supplementary Figure S5), but not in p53-null H1299 or p53^R273H^-mutant H1975 cells (Figure 5B). This suggests that F-LT1-3-PEG can enhance the efficacy of cisplatin against most p53 mutant lung cancers. For those cells do not express functional p53, inhibition of JNK activity restored the enhanced inhibitory effects of combined cisplatin and F-LT1-3-PEG treatments, as demonstrated by the proliferation of p53-null H1299 cells (Figure 5C).

### 3.12. Robo Receptors Are Not Involved in F-LT1-3-PEG’s Inhibition of Lung Cancer Cell Proliferation and Invasion

**Figure 6:**
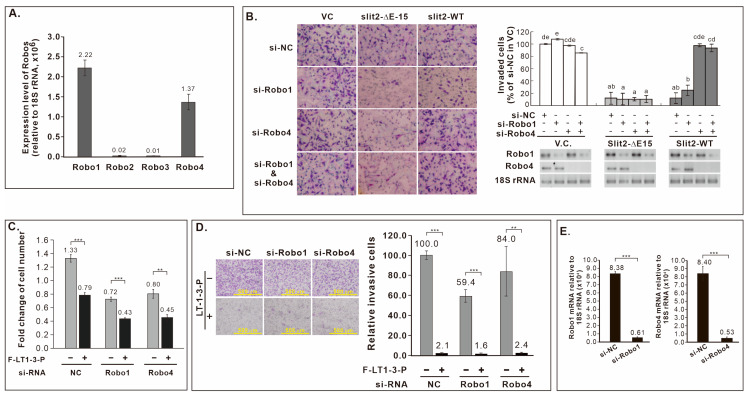
Role of Robo receptors in Slit2-WT- or Slit2-ΔE15-mediated inhibition of cell invasion and F-LT1-3-PEG-mediated inhibition of cell proliferation and invasion. (**A**) *Robo* mRNA expression in CL1-5, measured by real-time RT-PCR. (**B**) Knockdown of *Robo4*, but not *Robo1*, abolishes Slit2-WT-mediated invasion inhibition. Neither Robo1 nor Robo4 is involved in Slit2-ΔE15-mediated invasion inhibition. (**C**,**D**) Knocking down *Robo1* or *Robo4* does not abrogate the inhibitory effect of F-LT1-3-PEG on (**C**) proliferation or (**D**) invasion. (**E**) Knockdown efficiency of si-*Robo1* and si-*Robo4*, measured by real-time RT-PCR. Data are mean ± SD. Statistical significance was determined using a two-tailed unpaired Student’s *t* test; or one-way ANOVA with Scheffe’s multiple comparison test (**B**). Data points with the same letter are not significantly different. ** *p* < 0.01, *** *p* < 0.001.

Both Slit2-WT and Slit2-ΔE15 inhibited CL1-5 cell invasion, leading us to explore the involvement of known Slit2 receptors, Robo receptors, in this inhibitory effect. Given the expression of *Robo1* and *Robo4* in CL1-5 cells (Figure 6A), we knocked down *Robo1* and/or *Robo4* in CL1-5 cells stably expressing *Slit2*-WT or *Slit2*-ΔE15 for invasion assays. Knockdown *Robo4*, but not *Robo1*, reversed the inhibitory effect of *Slit2*-WT on invasion, while *Robo1* or *Robo4* knockdown did not eliminate the inhibited invasion phenotype of *Slit2*-ΔE15 cells (Figure 6B). As LT1-3 is part of LamG domain in both Slit2-WT and Slit2-ΔE15, we assessed whether Robo receptors mediate LT1-3’s inhibitory effect on proliferation and invasion. Knockdown *Robo1* or *Robo4* did not abrogate the inhibitory effect of F-LT1-3-PEG’s on cell proliferation (Figure 6C) or invasion (Figure 6D). Knockdown efficiency of si*-Robo1* and si-*Robo4* was confirmed via RT real-time PCR (Figure 6E). These results indicate that Robo receptors are not involved in F-LT1-3-PEG-mediated inhibition of lung cancer cell proliferation and invasion.

## 4. Discussion

In a previous study, we identified *Slit2*-WT and *Slit2*-ΔE15 splicing variants and demonstrated that *Slit2*-ΔE15 can inhibit both the proliferation and invasion of lung cancer cells, while *Slit2*-WT only possesses invasion inhibitory activity [22]. In this study, we identified F-LT1-3, a peptide derived from the Slit2-LamG domain, which exhibits dual roles in inhibiting the proliferation and invasion of lung cancer cells but not in inhibiting the proliferation of normal cell types. F-LT1-3 enhances the efficacy of cisplatin, a commonly used chemotherapeutic drug, and reduces cisplatin-induced side effects.

Despite advances in targeted therapy, a substantial proportion of NSCLC patients lack driver gene mutations [27,28]. Moreover, the combination of first-line immunological checkpoint inhibitors with platinum doublet chemotherapy has improved overall survival [29]. Therefore, platinum-based doublet chemotherapy regimens remain a cornerstone for patients without driver mutations or those resistant to targeted therapy. Given that F-LT1-3-PEG enhances cisplatin cytotoxicity and potentially reduces its side effects, it is a promising candidate for first-line lung cancer treatment in combination with cisplatin and continued maintenance therapy.

The Slit2/Robo signaling pathway has been implicated in both tumor suppressing and tumor-promoting roles, depending on the tumor type. Slit2 can undergo proteolytic cleavage into Slit2-N and Slit2-C fragments. The well-established axon guidance function of Slit2 primarily involves full-length Slit2 and Slit2-N, mediated by Robo receptors [10]. The LRR2 (D2) domain in Slit2 is critical for interacting with the Ig1 and Ig2 domains in Robo1, whereas Slit-C does not bind to Robo receptors [11,30,31]. Both Slit2-WT and Slit2-ΔE15 inhibit lung cancer cell invasion, but Robo4 is essential for Slit2-WT-mediated invasion inhibition in CL1-5 cells, while neither Robo1 nor Robo4 is required for Slit2-ΔE15-mediated invasion inhibition. These findings suggest distinct pathways of invasion inhibition for Slit2-WT and Slit2-ΔE15. LT1-3, derived from the Slit2-C region, maintains its inhibitory effects on proliferation and invasion even after knockdown of *Robo1* or *Robo4*.

Recent studies have uncovered novel functions of Slit2-C and a new Slit receptor, Plexin A1, in Slit2-mediated axon repulsion from the floor plate [32]. However, knocking down *PlexinA1* expression did not diminish F-LT1-3-PEG’s inhibitory effects on proliferation or invasion (Supplementary Figure S6). Therefore, F-LT1-3-PEG-mediated inhibition of cancer proliferation and invasion operates independently of Robo receptors, thereby exerting anti-proliferation and anti-invasion effects without activating potential oncogenic functions of the Slit/Robo axis. While our findings indicate that LT1-3 acts independently of known Slit receptors, the key cell surface targets that initiate the anticancer effects of LT1-3 remain to be identified in future studies.

*TP*53 mutations are frequently observed in cancers, associated with chemotherapy resistance [33,34,35], and linked to a poor prognosis [36,37,38]. We found that p53 positively regulates F-LT1-3-PEG-mediated inhibition of cell proliferation. Interestingly, most p53 mutants retained their ability to mediate F-LT1-3-PEG’s inhibitory effect, except for p53 R273 mutants. The R248 and R273 residues are situated on the loop of the DNA-binding domain, where R248 interacts with the DNA minor groove, and R273 interacts with the DNA backbone in the major groove [39]. Certain R248 and R273 mutations lead to gain-of-function effects. However, different mutations of the same residue may result in the acquisition of different phenotypes. For example, R248Q is associated with a more prominent phenotype than R248W [40]. R273H and R273C are gain-of-function mutations, while R273G is a loss-of-function mutation [41]. We found that both R248Q and R248W mediated the proliferation-inhibiting activity of F-LT1-3-PEG (Supplementary Figure S7), while none of the R273H, R273C, R273G, or R273W mutations mediated this activity. Thus, R273 residue in p53 is critical for F-LT1-3-PEG function.

Interestingly, a transcriptional suppressor of p53^R273H^, S240R, was able to restore cell sensitivity to F-LT1-3-PEG-mediated growth inhibition, suggesting downstream targets of p53 play an important role in the growth inhibition function of F-LT1-3-PEG. Therefore, comparing target genes downstream of various p53 mutations may shed light on the mechanism underlying the function of p53 in F-LT1-3-PEG-mediated proliferation inhibition. The expression pattern of these key target genes could serve as an indicator of the effectiveness of F-LT1-3-PEG in inhibiting cell proliferation.

The p53-reactivating compound Eprenetapopt (APR-246) has been shown to stabilize the p53 structure and restore wild-type p53 functions in p53-mutated cells [24]. A phase Ib/II study demonstrated that combination therapy with APR-246 and the hypomethylating agent azacitidine led to high clinical response rates and good tolerance in patients with myelodysplastic syndromes [42]. In H1975 cells expressing R273H mutation, APR-246 is able to restore cell sensitivity to F-LT1-3-PEG-mediated growth inhibition. Therefore, a combination therapy of F-LT1-3-PEG and APR-246 may be applicable to patients with certain p53 mutations.

**Figure 7:**
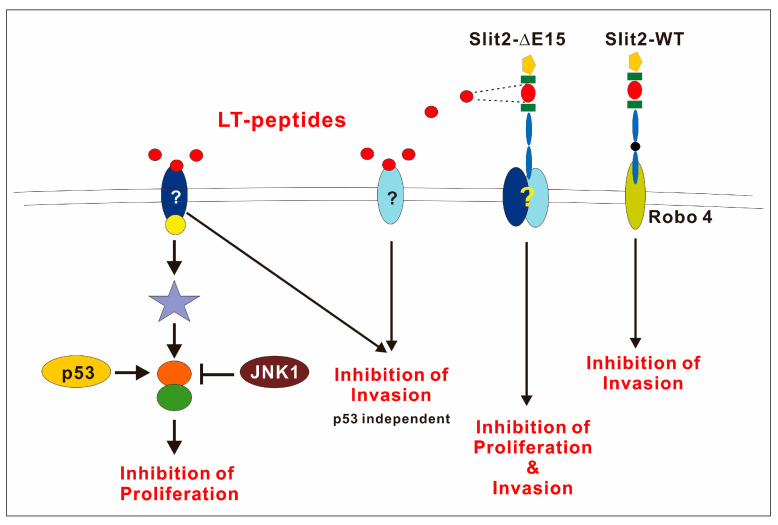
The schematic diagram shows that Slit2-WT inhibits cell invasion through Robo4. However, the receptor involved in Slit2-ΔE15’s inhibition of cell growth and invasion remains to be identified. p53 plays a positive role in regulating the growth inhibitory activity of the LT1-3 peptide, while JNK1 acts as a negative regulator.

The negative role of *JNK1* in F-LT1-3-PEG-mediated proliferation inhibition was evident, as JNK1 inhibition enhances growth inhibition or converts resistant cancer cells with p53 mutations to a sensitive phenotype. JNK and p53 crosstalk in many ways in response to stress, DNA damage, and apoptosis. JNK1 can bind and phosphorylate p53, which stabilizes p53 and promotes its function [43]. Alternatively, JNK downstream transcription factors (e.g., c-Jun, JunD) can positively or negatively affect p53 transcriptional activity [44,45]. However, we found that blocking JNK1 enhances growth inhibition effects of LT1-3, regardless of p53’ function in lung cancer cells. Therefore, we hypothesized that JNK1 and p53 regulate the crosstalk molecule oppositely; p53 positively regulates the target protein, whereas JNK1 negatively regulates the same target (Figure 7). This crossroad effector may serve as a potential biomarker for patient selection.

Cisplatin is a first-line chemotherapeutic drug for various cancers, including lung cancer. However, cisplatin often causes severe side effects, and p53 mutations can confer resistance to cisplatin. We found that F-LT1-3-PEG enhances cisplatin efficacy in lung cancer cells with p53 mutations and appears to reduce cisplatin-induced side effects in mice. Further studies are warranted to explore the mechanisms systematically underlying these beneficial effects. These findings suggest that F-LT1-3-PEG in combination with cisplatin may be a promising first-line therapy for lung cancer patients, including those with p53 mutations.

## 5. Conclusions

In conclusion, our study demonstrates that F-LT1-3-PEG is a multifunctional anti-lung cancer peptide with several desirable properties: it inhibits lung cancer cell proliferation and invasion, enhances cisplatin efficacy, reduces cisplatin-induced side effects, and exhibits no toxicity toward normal cells. Mechanistically, p53 positively regulates F-LT1-3-PEG-mediated growth inhibition, whereas JNK1 and PKA serve as negative regulators. F-LT1-3-PEG alone may be suitable for maintenance therapy, and F-LT1-3-PEG in combination with cisplatin shows promise as a first-line therapy for lung cancer.

## 6. Patents

LT1-3 and F-LT1-3-PEG peptides have been granted patents in Taiwan, the United States, Japan, and Mainland China, and a European patent application is pending.

## Figures and Tables

**Table 1:** Growth and invasion inhibitory activity of F-LT1-3-PEG in lung cancer cells.

	A549	H460	H1355	H23	CL1-5	H661	H1299	H1975
P53	WT	WT	E285K	M246I	R248W	R158L ^a^	Null	R273H
kRas	G12S	Q61H	G13C	G12C	WT	WT	WT	WT
Growth inhibition	+	+	+	+	+	+	−	−
Invasion inhibition	+	+	+	NA	+	NA	+	NA

NA: not available. ^a^ H661 contains R158L homozygous mutation and S215I heterozygous mutation.

## Data Availability

This paper incorporates the original contributions presented in the study. For further inquiries, please contact the corresponding authors.

## Supplementary Materials

The supplementary material can be downloaded at https://www.mdpi.com/article/10.3390/cells14211654/s1: Figure S1: Effects of LT-3, LT-4, and LT-5 on cell proliferation; Figure S2: Replacing the N-terminal L-phenylalanine residue with D-phenylalanine abolished the invasion inhibiting activity mediated by LT1-3; Figure S3: Effects of PEG-modified LT1-3 on the proliferation and invasion of CL1-5 cells; Figure S4: Effects of pathway inhibitors on F-LT1-3-PEG-mediated proliferation-inhibiting activity; Figure S5: F-LT1-3-PEG enhances the cytotoxicity of cisplatin in H23, H460, and H661 cells; Figure S6: The role of PLXNA1 in the growth and invasion of inhibitory activity of F-LT1-3-PEG; Figure S7: Effects of 53R248 mutants and the M245I mutant on F-LT1-3-PEG-mediated proliferation-inhibiting activity; Table S1: si-RNA target sites; Table S2: Primers for PCR-based mutagenesis; Table S3: Primers used for real-time PCR; Table S4: Peptide sequence.

## Abbreviations

The following abbreviations are used in this manuscript:

EMP	Epithelial–mesenchymal transition
Fmoc	Fluorenylmethyloxycarbonyl
NSCLC	Non-small cell lung caner
PEG	Polyethylene glycol
Q-RT-PCR	Quantitative reverse transcription polymerase chain reaction
