# Supplementary material for: LT1-3, a Slit2-Derived Peptide, Exhibits Anti-Tumor Activity and Improves Cisplatin Therapy
Source: Cells. 2025 Oct 22;14(21):1654. doi: 10.3390/cells14211654 (PMC12607519; doi:10.3390/cells14211654)
Supplement: Supplementary file 1 [file cells-14-01654-s001.zip › cells-3893080-supplementary.pdf]

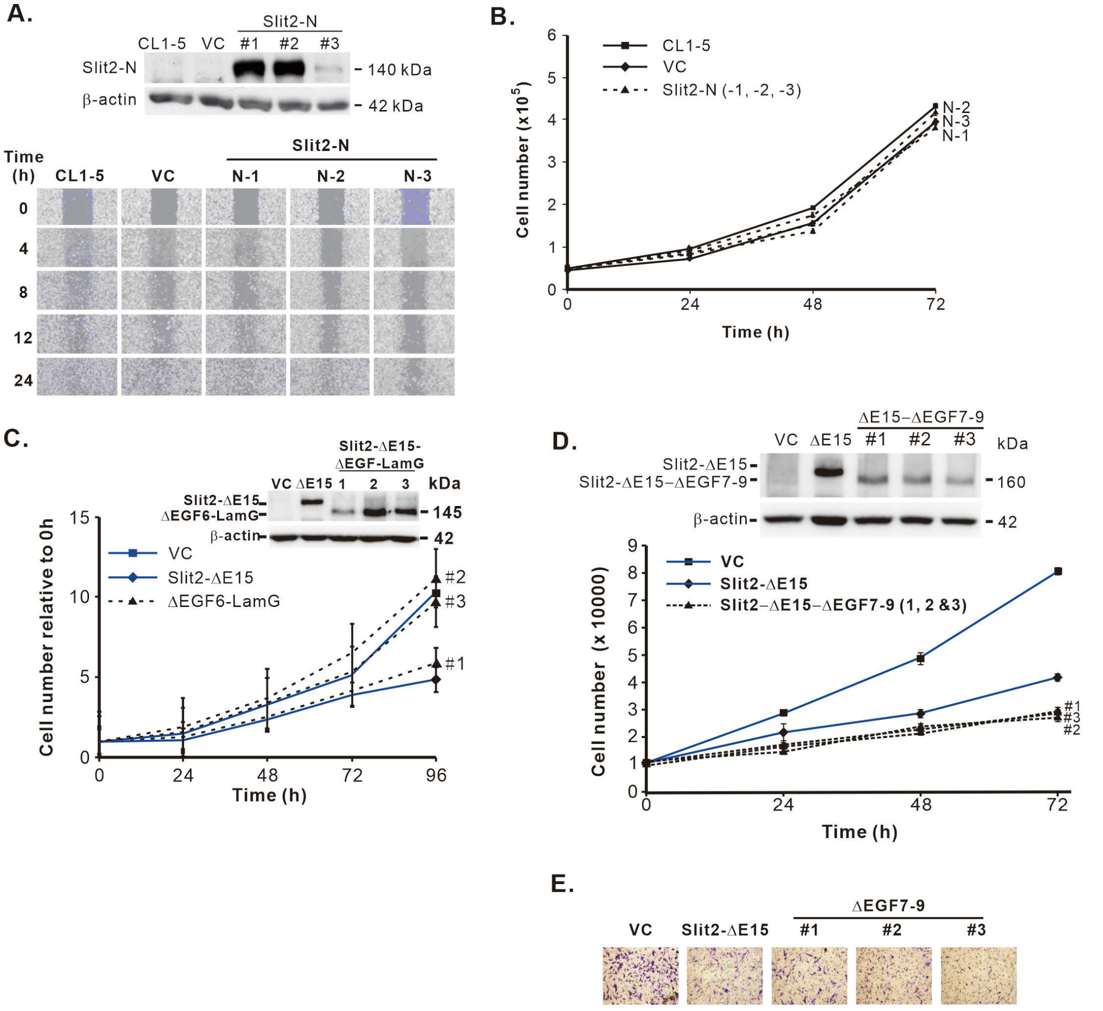

Figure S1. Effect of Slit2-N-terminal domain and Slit2-ΔEGF7-9 on cell proliferation and invasion. CL1-5 cells stably expressing the Slit2-N-terminal domain did not inhibit cell invasion (A) and proliferation (B). (C) Two stable clones of Slit2-ΔE15 lacking EGF6-LamG lost of growth inhibitory activity. Deletion of the EGF7-9 domain in Slit2-ΔE15 affected neither proliferation (D) nor invasion (E).

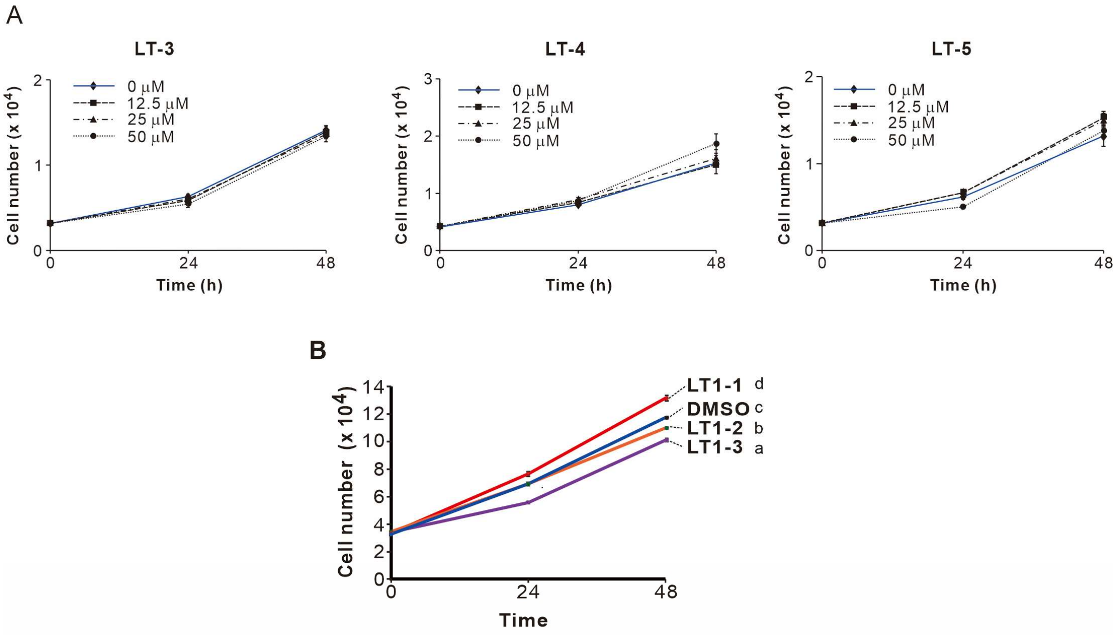

Figure S2. (A) LT-3, LT-4 and LT-5 did not inhibit CL1-5 cell proliferation. (B) LT1-3, but not LT1-1 and LT1-2, has the ability to inhibit cell proliferation.

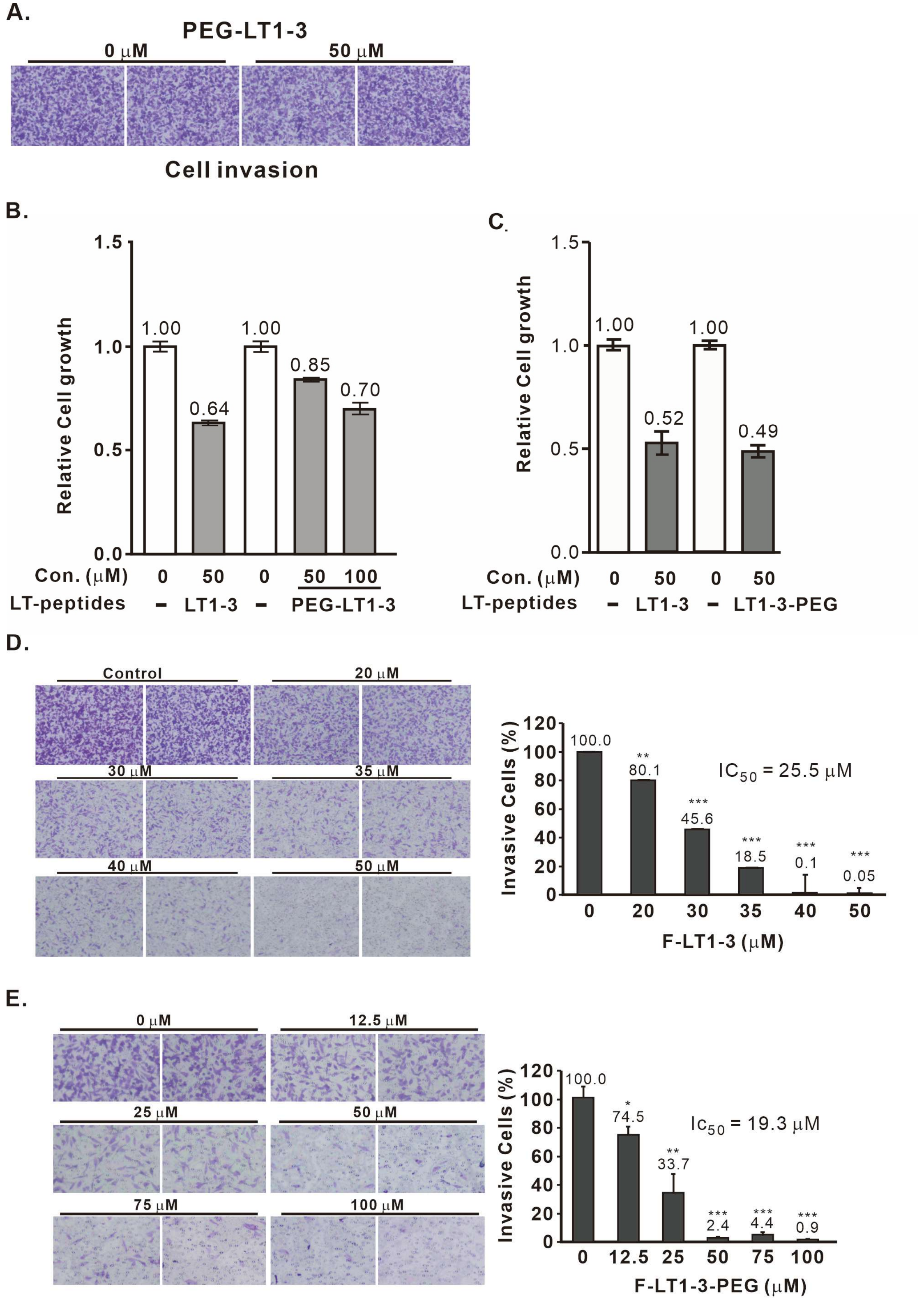

Figure S3. Effects of PEG-modified LT1-3 on the proliferation and invasion of CL1-5 cells. Addition of the N-terminus of PEG abolished the invasion inhibitory activity (A) and the proliferation inhibitory activity (B) of the LT1-3 peptide. (C) Modification of F-LT1-3 with PEG at its C-terminus (F-LT1-3-P) retained the proliferation-inhibiting activity of the peptide. (E) The F-LT1-3-PEG peptide had similar invasion inhibitory activity to that of the F-LT1-3 peptide.

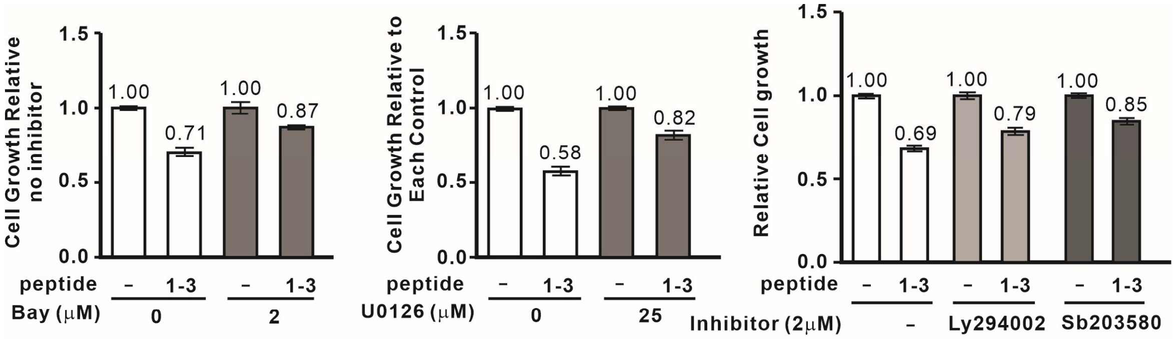

Figure S4. Effects of pathway inhibitors on F-LT1-3-PEG-mediated proliferation-inhibiting activity. An NFkB inhibitor (Bay11-7082), a MEK inhibitor (U0126), a PI3K inhibitor (Ly294002) and a p38 inhibitor (Sb203580) reduced the proliferation-inhibiting activity of F-LT1-3-PEG in CL1-5 lung cancer cells.

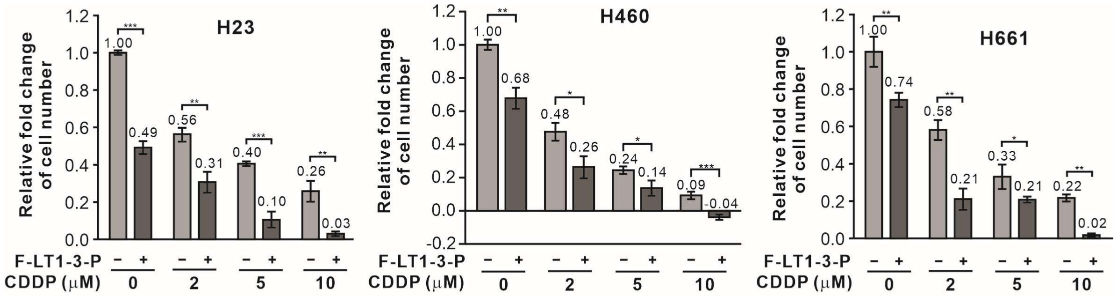

Figure S5. F-LT1-3-PEG enhanced the cytotoxicity of cisplatin in H23, H460 and H661 lung cancer cells.

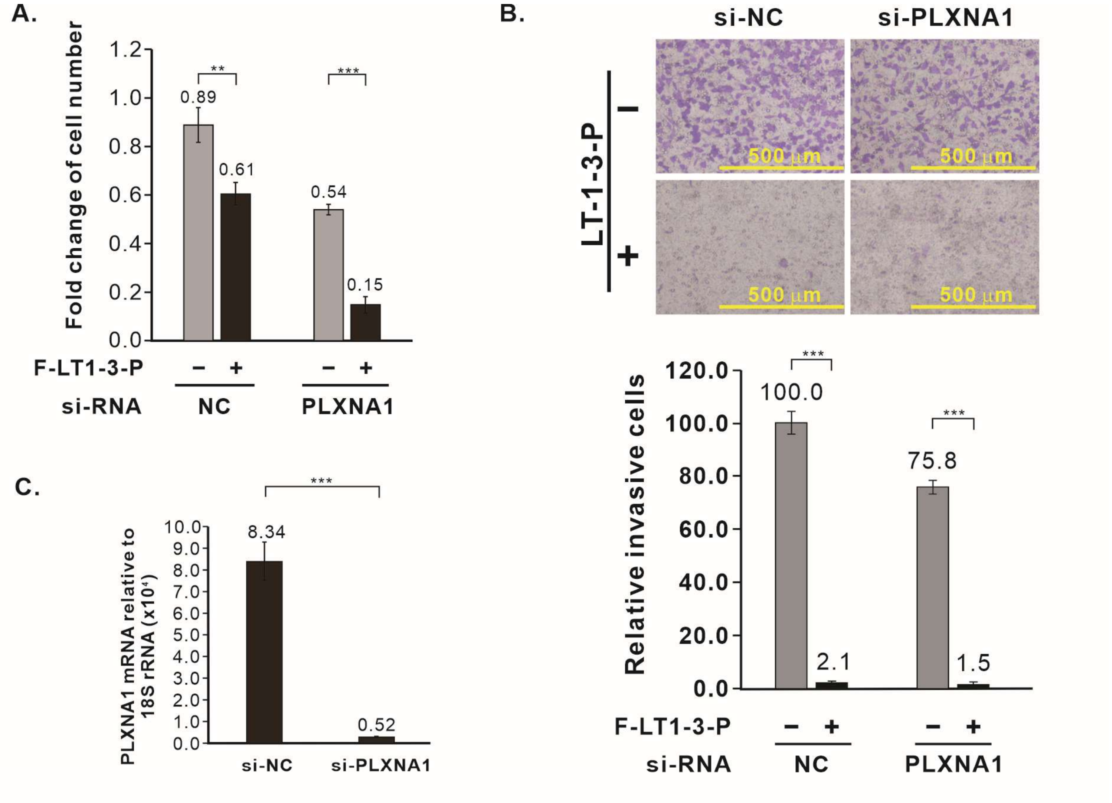

Figure S6. The role of PLXNA1 in the growth and invasion inhibitory activity of F-LT1-3-PEG. (A) Knocking down the expression of PlexinA1 did not abrogate the proliferation-inhibiting activity mediated by F-LT1-3-PEG. (B) Knocking down PlexinA1 expression also did not eliminate the inhibitory effect of F-LT1-3-PEG on cell invasion. (C) Estimated knockdown efficiency of PLXNA1 by Q-RT-PCR.

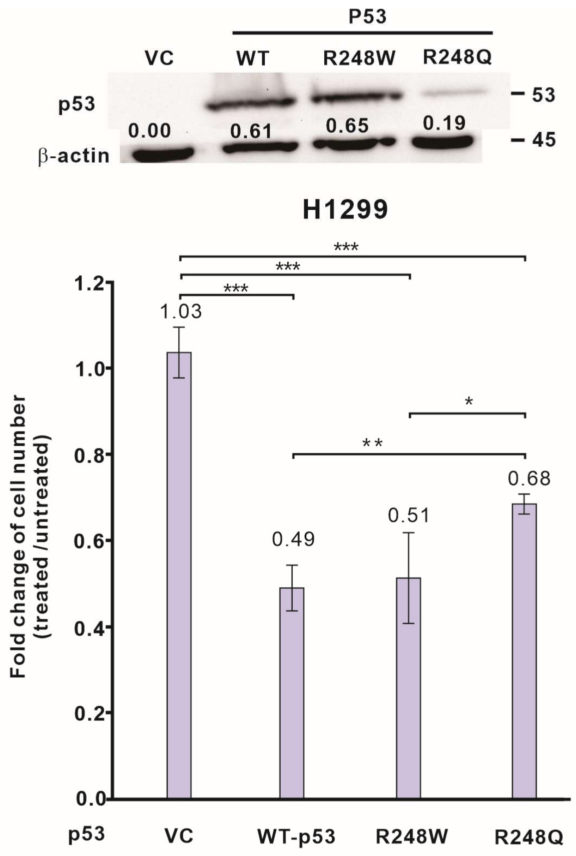

Figure S7. Effects of p53 R248 mutants and the M246I mutant on F-LT1-3-PEG-mediated proliferation-inhibiting activity. Wild-type p53, R248W or R248Q p53 mutant was transfected into H1299 cells, and these cells were treated with/without F-LT1-3-PEG. The relative change in the number of cells is reported as the fold change and was determined by the fold change in the number of treated cells divided by the fold change in the number of untreated cells. One-way ANOVA and the Scheffe test were performed to determine the relative fold change in the number of cells (treated/untreated) in the presence of p53. $^{*}p < 0.05$, $^{**}p < 0.01$, $^{***}p < 0.001$

Supplementary Table S1. si-RNA target sites

Gene	siRNA sequence
si-*Rbobo1*	GCAGACACGTGGCCTAATA
si-*Rbobo4*-1	GCTTCTGGCTGTGCGAATT
si-*Rbobo4*-2	CCAAGACTACGAGTTCAAA
si-*p53*	GACTCCAGTGGTAATCTAC
si-*PKA*ca	GCAGGAGAGCGTGAAAGAA
si-*JNK1*	CCAACACCCGTACATCAAT

Supplementary Table S2. Primers for PCR-based mutagenesis.

Name of mutation	Forward primer	Reverse primer
Slit2-N	pcDNA3.1-myc/His (-)<br>5′-GTAGGCGTGTACGGTGGGA-3′	Slit2-1118R-KpnI<br>5′-GGGGGTACCGACCATGGGTGGAGAAAA-3′
Slit-C	Slit2-1119F-EcoRI<br>5′-CCGGAATTCCCTCCCTCGTACCAGCCC -3′	Slit2-4791R-kpnI<br>5′-GGGGGTACCGGACACACACCTCGTACAG-3′
◎EGF6-LamG	◎EGF6-LamG-F<br>5′-TCGTACCGAGCCATGCCACAA-3′	◎EGF6-LamG-R<br>5′-ATGGCTCGGTACGAGGGAGGAC-3′
◎EGF7-9	◎EGF7-9-F<br>5′-TGGCTGTTGCAGCAGTGGATACA-3′	◎EGF7-9-R<br>5′-GCTGCAACAGCCAGGCAAAAT-3′
LamG-F	LamG-F-RsrII<br>5′-CCGCGGTCCGACCTCAGACGAACATAACACT-3′	LamG-F-RsrII<br>5′-CCGCGGACCGCCTCACTGTTGATGTAAAGGTT-3′
LamG-C	LamG-C-RsrII<br>5′-CCGCGGTCCGAGCTTCTGCCATTTACAGTGT-3′	LamG- F –RsrII<br>5′-CCGCGGACCGCCTCACTGTTGATGTAAAGGTT-3′
Vector primer for PCR-based mutagenesis	pcDNA3.1-myc/His (-) /<br>F′-upstream primer (F′-up)<br>5′-GTAGGCGTGTACGGTGGGA-3′	pcDNA3.1-myc/His (-) /<br>R′-downstream primer (F′-dp)<br>5′-CTAGAAGGCACAGTCGAGG-3′

Supplementary Table S3. Primers used for real-time PCR.

Gene	Forward primer	Reverse primer
Robo1	5′-AGCCTCGCTCAGCAGATT-3′	5′-TGACAGCTTCGCCTCCTCT-3′
Robo4	5′-CGGCTGTCTGTGGCTGT-3′	5′-CCCTTCGTCACTCTTCTCTG-3′
p53	5′-GCGTGTGGAGTATTTGGA-3′	5′-GAGAGGAGCTGGTGTTGTT-3′
JNK1	5′-CCCTGATGTCCTTTTCCC-3′	5′-<br>CCCTTTCATCTAACTGCTTGTC-3′
PKA	5′-GCCGCCGCCAAGA-3′	5′-ATCACCCGCCCGAA-3′
Plexin A1	5′-AGCGGGTGGTGAAACTCTA-3′	5′-ACACGGCCCTCCAGGAA-3′

Supplementary Table S4. Peptide sequence

Name	Sequence
91AA	ASAIYSVETINDGNFHIVELLALDQSLSLSVDGGNPKIITNLSKQSTLNFDSPLYV<br>GGMPGKSNVASLRQAPGQNGTSFHGCIRNLYINSE
LT-1	ASAIYSVETINDGNFHIVELLA
LT-3	KIITNLSKQSTLNFDSPLYVGG
LT-4	GGMPGKSNVASLRQAPGQNGTSF
LT-5	LRQAPGQNGTSFHGCIRNLYINSE
LT1-1	ASAIYSVETI
LT1-2	ETINDGNFHI
LT1-3	FHIVELLA
